# Learning outcomes from a biomedical research course for second year osteopathic medical students

**DOI:** 10.1186/1750-4732-4-4

**Published:** 2010-07-08

**Authors:** des Anges Cruser, Sarah K Brown, Jessica R Ingram, Alan L Podawiltz, Bruce D Dubin, John S Colston, Robert J Bulik

**Affiliations:** 1 University of North Texas Health Science Center, Texas College of Osteopathic Medicine, Fort Worth, Texas, USA; 2 Rocky Vista University College of Osteopathic Medicine, Parker, Colorado; 3 University of Texas Medical Branch, Galveston, Texas, USA

## Abstract

**Background:**

A ubiquitous dilemma in medical education continues to be whether and how to integrate research competencies into the predoctoral curriculum. Understanding research concepts is imbedded in the six core competencies for physicians, but predoctoral medical education typically does not explicitly include research education. In an effort to quickly report academic research findings to the field, this is the second in a series of articles reporting the outcomes of a research education initiative at one college of osteopathic medicine. The first article described the competency model and reported baseline performance in applied understanding of targeted research concepts. This second article reports on the learning outcomes from the inaugural year of a course in basic biomedical research concepts.

**Methods:**

This course consisted of 24 total hours of classroom lectures augmented with web-based materials using Blackboard Vista, faculty moderated student presentations of research articles, and quizzes. To measure changes in applied understanding of targeted research concepts in the inaugural year of the course, we administered a pretest and a posttest to second year students who took the course and to first year students who took an informatics course in the same academic year.

**Results:**

We analyzed 154 matched pretests and posttests representing 56% of the 273 first and second year students. On average, the first year (53) and second year students (101) did not differ in their mean pretest scores. At posttest the second year students showed significant improvement in their applied understanding of the concepts, whereas the first year students' mean posttest score was lower than their mean pretest score.

**Conclusions:**

This biomedical research course appears to have increased the second year students' applied understanding of the targeted biomedical research concepts. This assessment of learning outcomes has facilitated the quality improvement process for the course, and improved our understanding of how to measure the benefits of research education for medical students. Some of the course content and methods, and the outcome measures may need to be approached differently in the future to more effectively lay the foundation for osteopathic medical students to utilize these concepts in the clinical setting.

## Background

A recent historical review of how physician-scientists and clinical researchers have trained for their work recommends increasing research content in the medical school curriculum [1]. The National Institutes of Health support initiatives under a number of programs to increase early career exposure to research for medical students, including the Research Education Project Partnership R25 Grant mechanism supporting this project. Regardless of the perceived value of early career research education, a ubiquitous dilemma remains as to how to incorporate biomedical research competencies into the predoctoral curriculum. Medical schools focus on training clinical practitioners generally reserving research education for dual-degree students. Residency programs however, are placing increasing emphasis on research competence while current evidence suggests that new graduates continue to be research-naïve and underprepared for the biomedical research expectations of their post graduate programs [2].

As residency programs expand their expectations for research participation, and students are increasingly expected to apply evidence based medicine (EBM) principles in their clinical rotations, research competencies may become essential rather than optional. Research, EBM and statistics are of necessity interrelated, and to meaningfully navigate the medical research literature, practicing physicians may benefit from an applied, conceptual grasp of research concepts more than from a quantitative statistical rubric [3,4]. In fact, EBM experts endorse research literacy to improve critical thinking and enhance clinical decision making [5]. Thus we suggest that medical students may benefit in many important ways from acquiring biomedical research competencies early in their medical education.

Regardless of the theoretical value of including research content in the medical school curriculum, there are numerous obstacles to its implementation. For example, the six core competencies required in post graduate medical education and questions in national licensure exams tend to focus on understanding the statistics of research versus understanding research design suitable to answer a particular study question. Also, the methods traditionally used to instruct medical students in research outside of a dual-degree track have included short statistics courses, or elective experiences in bench or clinical research labs. Interestingly, the literature indicates that statistics courses and brief immersion in on-going research are among the least recommended methods for effectively preparing future physicians to be proficient in the language of biomedical research [6].

While methods for medical education are evolving to more sophisticated teaching paradigms, increasing efficiencies with electronic technology and assessing more than knowledge outcomes such as measures of critical thinking skills, these trends tend to occur more as evolutions, not revolutions. To rapidly impact the dearth of clinical researchers nationally we may need a revolution in how we define research competencies for medical students. The hypothesis is that that early career familiarity with the world of biomedical research at a minimum can improve critical thinking skills, better prepare students for expectations of residency training programs, and improve performance on national exams. Teaching research concepts only to answer questions on an exam may miss important opportunities to inspire early career physicians in the language of research.

It has been generally accepted that biomedical research competencies for physician scientists develop along a continuum, ideally beginning with early career exposure to research concepts, extending through post-doctoral research training, culminating in the quintessential practice of research and conducting independent multi-center clinical trials [7]. This suggests that a multi-tiered competency model may help differentiate among basic, intermediate, and advanced research competencies [8]. Using the three-tiered model we have previously described, we can define competencies as targeted learning outcomes.

The three tiers of this model can be briefly defined in the following way. Tier-one competencies represent a basic, foundational understanding of research. A tier-one individual would be a proficient professional consumer of biomedical research information. Tier-two competencies are associated with an intermediate research realm such as a master's degree or a predoctoral research track in which the individual conducts mentored research. Tier-three encompasses advanced research skills acquired in a dual-degree doctoral program or a post-doctoral clinical research fellowship.

Tier-one appears to be the most suitable competency level for research-naïve medical students because it targets a basic, foundational understanding of research language and emphasizes applied understanding of commonly used biomedical research concepts. These concepts are those most often tested in national exams and most frequently used in the medical literature [4]. The tier-one competencies are the learning objectives for the biomedical research course we taught to second year students. The course utilized multiple methods including traditional classroom lectures, reading assignments in selected texts [3,4], assigned articles for critical review, web-based and on-line resources, and dialogs with expert faculty.

All articles assigned to the students corresponded to the medical topic or system the students were studying at that time. To critique the articles, students used specified guidelines and completed templates guiding them through the critique process. Students also had access to classroom lectures and Blackboard Vista slides addressing the research concepts contained in each article. Small groups of students each presented a critique to their classmates and expert faculty, with slides and lecture materials of their own development. The templates are two *data sheets *provided as Additional file 1: Appendix I to this article. The year-long course was integrated into the clinical medicine component of the second year and represented 10% of that course grade. Students were graded on the content of their templates, the accuracy of their presentations, and periodic quizzes.

Several studies have reported residents' and practicing physicians' understanding of and attitudes toward biostatistics [2,9,10] but we found no published studies regarding second year US medical students' understanding of biomedical research concepts. In an effort to communicate academic research findings to the field in a timely manner, this article reports on the learning outcomes from the inaugural year of the course during the academic year 2008-09.

We have presented the results of the pretests and posttests of applied understanding of research concepts for the second year students who took the course, and the results of the same pretests and posttests for first year students who took a basic informatics course during the same academic year. The informatics course focused on search methods and case-studies presentations. While students in the informatics course included at least one research article in their search to develop their case presentations, they were not instructed on research concepts in that course. No pretests or posttests were administered to measure the outcomes of that course independently from this study. We have also provided the 20-item test as Additional file 1: Appendix II to this article.

## Methods

With the support of an NIH R25 grant and a grant from the Osteopathic Heritage Foundation, a 20-item test was developed by a panel of biomedical researchers and academic physician faculty at the medical school. The initial list contained 50 items taken from published studies of research competencies, national databases, and original questions developed by local research and physician scientists [8,2,11]. The final 20 items emerged from a series of content reviews by the project advisory committee, beta-testing by clinical and research faculty and pilot testing with 12 volunteers from both classes. Selection of final questions was guided by the principle that tier-one competencies should focus on applied understanding of the most frequently encountered research concepts in the medical literature [3,4]. Thus the questions were framed in case contexts as much as possible. Seven of the final 20 items originated with permission from Windish [2], and 13 items were locally developed.

Each final question presented four choices with one preferred correct answer, and a no-response (NR) option. We chose to include a NR option to examine several dimensions of the learning outcomes and examine the value of this form of testing. Attempting to measure research readiness, Windish reported that medicine residents did not perform well on a similar test [2]. Our project advisory committee endeavored to match the challenge level of each question with expectations they had of predoctoral students at a tier-one level, and reproduce as much as possible the types of questions students might encounter on national board exams.

We asked, with IRB approval, all 335 enrolled first (177) and second (158) year medical students to take the test at the beginning (July) and end (May) of academic year 2008-09. Students completed the 20-item test on-line using the school's secure on-line testing web site with the course director present. Students were instructed to attempt to answer only the questions for which they believed they knew the correct choice.

Questions on the course quizzes were associated with the same competencies covered in the 20-item pretest and posttest but did not contain an NR, and were directly linked to the four to five articles critiqued in the class period immediately previous to the quiz. Brief recaps of each student presentation were provided by faculty immediately following the presentation, reviewing the research concepts contained in that article that would be covered on the next quiz. The underlying philosophy of the course was that we wanted students to learn the material and succeed.

Data were compiled using the school's secure on-line testing platform and exported to an excel file. Academic services provided demographic data and matching for pretests and posttests. Data were exported to an SPSS file for analysis. Analyses included calculating proportions of correct responses for each of the 20 items and corresponding confidence intervals, and t-Tests and Chi-Square tests to examine differences between groups for demographics and performance scores.

## Results

There were 273 (81%) students who completed the posttest. Among these 273 students seven were dropped from the analysis because of having marked all NR choices on their posttests, although they had answered some questions on their pretests. After excluding these students and matching all remaining students who had complete data on both pretest and posttest questionnaires, we retained 154 students (56%) in the final analysis. Among the 154 students in this sample, 101 were second year and 53 were first year students representing 65% and 34% of each class respectively.

According to the data reported nationally by this school, the gender, racial, and ethnic composition of all medical students in 2008 was 54% males and 46% females; 48% White, 6.5% Hispanic, 19% Asian, and 2.7% Black. Academic services provided the race and gender data for the students in this sample reporting 55% (85) were males and 45% (69) were females; 59.7% (92) of the sample were White, 9.7% (15) Hispanic, 23.4% (36) Asian, and 5.2% (8) Black. Thus the sample is comparable to the student body.

In our baseline analysis we assessed the impact of pre-enrollment degree status on test scores. Thus we report that among these 154 students in this sample 112 (73.7%) had a pre-enrollment bachelor's degree only, and 40 (26.3%) had a pre-enrollment master's or doctoral degree, and two were missing complete information. Using the highest reported MCAT score for each student, the average MCAT score for this sample was 27.49 (SD 3.08). Chi-square and *t *tests examining gender, race, ethnicity, previous education and MCAT scores found no significant differences between the two classes.

In order to determine if second year students improved their applied understanding of the targeted research concept, we used the proportion of correct answers out of all 20 questions. In this study sample of 154 students the overall mean pretest score out of a possible 100, was 28.90 (SD 16.83; range, 0-70), and the mean posttest score was 35.58 (SD 18.35; range, 0-75). The two classes did not differ in the pretest scores (*t *_152 _= -1.56, *P *= .12), but at posttest the second year students scored significantly higher than the first year students in this sample (*t *_152 _= 4.25, *P *< .001).

We were primarily interested in whether second year students improved in their applied understanding of the targeted research concepts after the course. Table 1 displays the results of t-tests examining changes in scores from the pretests to the posttests for each class. There were three dimensions examined: the proportion of questions answered correctly out of all 20 items, the number of NR choices out of 20, and the proportion of questions answered correctly out of only those attempted (i.e. non NR). The two classes differed in all three dimensions.

**Table 1:** Differences in Pretests and Posttests for Second Year (N = 101) and First Year (N = 53) Students

		Pretest Mean	Posttest Mean	Mean Change	*P *value*
Percent Correct of 20	Second Year	27.4%	39.9%	12.5%	< .001
	First Year	31.8%	27.4%	-4.4%	
					
Number No Response of 20	Second Year	8.56	5.11	-3.46	< .001
	First Year	8.08	8.53	0.45	
					
Percent Correct of Attempted	Second Year	47.5%	54.4%	6.8%	.023
	First Year	51.3%	48.8%	-2.5%	

* *t *test for change in score between pretest and posttest

The second year students' overall average score improved by 12.5%, compared to the average decrease of almost 4.4% for the first year students *(t *_152 _= 5.54, *P *< .001). This change is the equivalent of an increase of 2.5 out of 20 questions answered correctly by the second year students, and almost one less question answered correctly at posttest by the first year students.

Because the frequency of NR choices could impact both the student's score and the percent of students responding correctly to each question, we also examined NR choices. NR was provided as a response option to encourage students to attempt only questions they believed they could answer without guessing. We found no difference between the two classes in the frequency of NR choices at the pretest, at 8.56 (SD 5.98) for second year students, and 8.08 (SD 5.50) for the first years (*t *_152 = _.495, *P *= .62). At the posttest however, second year students made significantly fewer NR choices with a mean of 5.11 (SD 5.53), compared to first year students who selected a mean of 8.53 (SD 5.91), (*t *_152 _= -3.56, *P *< .001). The change in number of NR responses from pretest to posttest reflects significant improvement (based on a reduced reliance on the NR choice) for the second year students compared to the first year.

To explore this observation further, we next considered how students performed relative to the number of questions out of 20 that they attempted (non NR). At pretest, the percent of correct answers for only the attempted questions was 47.5% for the second year students compared to 51.3% for the first year students. At posttest the second year students had significantly increased the proportion of correct answers out of the attempted questions with 54.4% correct. The first year class at posttest however, reflected a significant decrease in the percent of correct answers out of the attempted questions to 48.8% *(t *_152 _= 2.30, *P *= .02).

Last we examined performance on each of the 20 items. Table 2 displays the pretest and posttest results and its corresponding 95% confidence interval for each item by class using the percent of correct responses for each of the 20 questions. Items are labeled to facilitate the reader's association with the test questions provided as an appendix to this article.

**Table 2:** Percentages of Correct Pretest and Posttest Answers Second Year (N = 101) and First Year (N = 53) Classes

	Research Concept	Class	Pretest	Posttest
			Correct (95% CI)	Correct (95% CI)
1	Statistical significance	Second Year‡	15.8 (8.7-23.0)	32.7 (23.5-41.8)
		First Year	15.1 (5.5-24.7)	20.8 (9.8-31.7)
2	Type II error	Second Year‡	30.7 (21.7-39.7)	54.5 (44.7-64.2)
		First Year	35.8 (22.9-48.8)	34.0 (21.2-46.7)
3	Sensitivity-Specificity	Second Year‡	51.5 (41.7-61.2)	71.3 (62.5-80.1)
		First Year	47.2 (33.7-60.6)	52.8 (39.4-66.3)
4	Phases of clinical trials†	Second Year‡	43.6 (33.9-53.2)	74.3 (65.7-82.8)
		First Year	43.4 (30.1-56.7)	37.7 (24.7-50.8)
5	Linear regression	Second Year	47.5 (37.8-57.3)	50.5 (40.7-60.2)
		First Year	52.8 (39.4-66.3)	49.1 (35.6-62.5)
6	Essential parts of published research	Second Year	57.4 (47.8-67.1)	53.5 (43.7-63.2)
		First Year	67.9 (55.4-80.5)	54.7 (41.3-68.1)
7	Protection of human subjects	Second Year‡	11.9 (5.6-18.2)	40.6 (31.0-50.2)
		First Year	13.2 (4.1-22.3)	13.2 (4.1-22.3)
8	Case-control design*	Second Year	3.0 (0.0-6.3)	6.9 (2.0-11.9)
		First Year	0.0 (0.0-0.0)	3.8 (0.0-8.9)
9	Interpretation of results	Second Year	21.8 (13.7-29.8)	33.7 (24.4-42.9)
		First Year	17.0 (6.9-27.1)	18.9 (8.3-29.4)
10	Define Bias*	Second Year	16.8 (9.5-24.1)	22.8 (14.6-31)
		First Year	22.6 (11.4-33.9)	18.9 (8.3-29.4)
11	Power and sample size *	Second Year	4.0 (0.2-7.8)	11.9 (5.6-18.2)
		First Year	15.1 (5.5-24.7)	13.2 (4.1-22.3)
12	Interpretation of descriptive statistics *	Second Year	35.6 (26.3-45.0)	45.5 (35.8-55.3)
		First Year	45.3 (31.9-58.7)	37.7 (24.7-50.8)
13	Chi-square statistic	Second Year	25.7 (17.2-34.3)	37.6 (28.2-47.1)
		First Year	32.1 (19.5-44.6)	17.0 (6.9-27.1)
14	Validity of results†	Second Year	68.3 (59.2-77.4)	70.3 (61.4-79.2)
		First Year	77.4 (66.1-88.6)	71.7 (59.6-83.8)
15	Positive predictive value	Second Year‡	14.9 (7.9-21.8)	34.7 (25.4-43.9)
		First Year	13.2 (4.1-22.3)	9.4 (1.6-17.3)
16	Continuous variables*	Second Year‡	12.9 (6.3-19.4)	30.7 (21.7-39.7)
		First Year	35.8 (22.9-48.8)	22.6 (11.4-33.9)
17	Ordinal variables*	Second Year	27.7 (19-36.5)	44.6 (34.9-54.2)
		First Year	24.5 (12.9-36.1)	24.5 (12.9-36.1)
18	Nominal variables*	Second Year	16.8 (9.5-24.1)	30.7 (21.7-39.7)
		First Year‡	32.1 (19.5-44.6)	9.4 (1.6-17.3)
19	Research ethics	Second Year	28.7 (19.9-37.5)	24.8 (16.3-33.2)
		First Year	20.8 (9.8-31.7)	22.6 (11.4-33.9)
20	Sample size calculations	Second Year	12.9 (6.3-19.4)	26.7 (18.1-35.4)
		First Year	24.5 (12.9-36.1)	15.1 (5.5-24.7)

* From Windish

† Two possible correct answers

‡ *P *< 0.05 for pretest and posttest differences

Referring to Table 2, at pretest performance was generally low for all 20 items. Only two questions were answered correctly by more than 50% of the students: item 6) *essential parts of published research*, and item 14) *validity of results*. From pretest to posttest, second year students improved their performance in 18 of 20 competencies with significant improvement in seven of those areas including *statistical significance, recognizing a Type II error, defining sensitivity and specificity, recognizing phases of clinical trials, human subjects' protection, positive predictive value of a test*, and *recognizing continuous variables*. Interestingly, the first year students exhibited significantly diminished performance in recognizing nominal variables. Students' performance was lowest in both pretest and posttest responses to item 8) *recognizing the research design *and item 11) *understanding power and sample size*.

In addition to the 20-item pretest and posttest second year students in the course completed seven quizzes during the academic year. Quiz questions assessed knowledge of the research concepts found in the research articles reviewed in the previous period. Average quiz scores for the second year class improved from 73.3% (range, 40 - 100) in the first semester to 86.8% (range, 41 - 100) by the end of the inaugural year of the biomedical research course. The second semester's mode score was 100 compared to the first semester mode of 73.

## Discussion

This study included students in the first and second year classes who had complete pretests and posttests at one osteopathic medical school. Only one class completed the research course; and although the students in this sample appear representative of all students in both classes, we do not know whether students whose scores were not included due to incomplete or unmatchable tests were different.

Also, the questions used to measure pretest and posttest applied understanding of the targeted concepts have not been validated. Our questions used both novel and published questions and attempted to target a tier-one level of applied understanding of research, a level of difficulty lower than that presumed to be suitable for graduate physicians [2].

Although the second year students improved their applied understanding of all but two targeted concepts, posttest performance in a number of areas remained low, with less than 50% of respondents answering the majority of questions correctly. This suggests that this course was not yet achieving its full potential.

We used the findings of this study to strengthen the course for its second year by augmenting lecture materials, increasing web-based resources, and strengthening guidelines to reinforce learning. In May 2010 these two classes will take the posttest at the end of the second year of teaching the course: the class of 2011 to examine retention, and the class of 2012 to measure their learning outcomes from the course. Following that assessment, the 20-item questionnaire, the course content and the teaching methods will undergo a formal academic quality review for possible future modifications.

## Conclusions

While the literature emphasizes the need to prepare future physician scientists to understand the relationship between EBM, statistics and research [12-18], national board exams continue to restrict measures of research competencies to very few questions. Beyond the argument that early-career exposure to biomedical research may improve critical thinking skills, there are other reasons to include biomedical research competencies in the medical school curriculum. For example, the Association of American Medical Colleges predicts that competition for limited residency slots will become keener [19], and residency training programs will likely continue to emphasize meaningful scholarly projects in research [20].

In a recent critical review of the history of clinical research training in the US, Teo makes eight powerful and empirically based recommendations [1]. Four of these well crafted recommendations pertain directly to the nature of the infrastructure of research education in medical schools. Teo recommends exposing medical students to concepts of clinical research as part of their educational curriculum, offering several different stages of research training opportunities equivalent to our tiered model, and placing emphasis on early career exposure to research.

As the second in a series of articles this paper reports improvements in second year medical students' applied understanding of targeted tier-one research concepts. The third article will provide the results of the second time the year long biomedical research course has been taught, focusing on the iterative nature of achieving these competencies and the tension between quantitative statistics learning and clinical research understanding. The next article will examine the application of these competencies in clerkship rotations, and the following article will report on our collaborations with other schools in examining learning outcomes from other models of research education.

Innovations in teaching biomedical research concepts have been reported as successful in British medical schools and other health professions training programs [6,21], but there is still very limited research on integrating research education into predoctoral medical education in the United States. Research competencies are included among the core competencies required for post graduate education. National accreditation bodies now require the colleges of osteopathic medicine to consider incorporating those competencies into the predoctoral curriculum as a minimum for meeting accreditation standards. If we hope to provide our osteopathic medical students with the highest possible quality biomedical research education, and inspire research-savvy osteopathic physician leaders, educators, and scientists, we need to reach beyond the minimum standards and inspire early career understanding and appreciation of research as a sine-qua-non of medicine. It is indeed advisable to have many models of brave new-world style policies and practices that firmly and clearly encourage and support early career research education [22].

If this method increases students' appreciation of research in medicine, enhances their life-long learning perspective, and also better prepares them for national licensure exams it will have achieved its goals. Improved research competencies also mean students will be better prepared for post graduate training research requirements.

## Competing interests

The authors declare that they have no competing interests.

## Authors' contributions

dAC led the development of the test questionnaire and developed the manuscript with the team. SKB performed the statistical analysis and contributed to the discussion and conclusions. JRI cleaned the data and contributed to the interpretation of the analysis. ALP, BDD, and RJB contributed to the crafting of the questions and the interpretation of the results. JSC participated in the course and applies concepts learned to his teaching responsibilities in the manipulative medicine predoctoral fellowship and contributed to the interpretation of the results.

All authors have read and approved the final manuscript.

## Supplementary Material

Additional file 1 **Appendices**. Appendix I and II. Click here for file
