# Supplementary material for: Learning outcomes from a biomedical research course for second year osteopathic medical students
Source: Osteopath Med Prim Care. 2010 Jul 8;4:4. doi: 10.1186/1750-4732-4-4 (PMC2914734; doi:10.1186/1750-4732-4-4)
Supplement: Additional file 1 — Appendices. Appendix I and II. [file 1750-4732-4-4-S1.PDF]

Appendix I Data Sheets

OVERVIEW PERSPECTIVE

1. Purpose: This study was conducted to (select one best response):

   ☐ Increase understanding of individual health status or a public health issue such as risk for disease, incidence or prevalence of a disease or event, or transmittal of a virus or bacteria).

   ☐ Increase our scientific understanding of a disease etiology or a treatment method.

   ☐ Examine the safety and efficacy of a treatment.

   ☐ Explain behaviors, opinions, procedures or other phenomena.

2. Study Type: Select the description(s) that best describes this study (not the study research design).

   ☐ Descriptive ☐ Existing data or using data in a novel way

   ☐ Survey or interviews ☐ Experimental ☐ Policy or services research

   ☐ Exploratory or pilot research ☐ Other (specify)

3. Sample: Complete each box for this study (you may use a zero or an NA as appropriate:

   Total Population → Screened or Identified → Eligible → Enrolled or Retained → Dropped or Lost → Final N for data analysis

4. Protocol:

   a. Did the study include control or experimental groups? (select all that apply):

      ☐ Treatment ☐ Placebo Control ☐ No-treat/standard care only control

      ☐ Condition Present ☐ Condition not Present ☐ Other (specify):

   b. Interventions (select all that apply):

      ☐ No Intervention or Treatment ☐ Drug/Phamaco-Therapy ☐ Manual Medicine

      ☐ Education ☐ Other CAM Modality or Treatment (specify):

      ☐ Other (specify):

   c. Primary Outcome(s) (Measurement or Variables up to two):

      1) 2)

   d. Data Sources (select all that apply):

      ☐ Interviews (open, structured, questionnaire) ☐ Existing Data Set

      ☐ Clinical Observation or Opinion Survey ☐ Distributed Survey

      ☐ Patient charts/records ☐ Other (specify)

   e. Any special methods or precautions used to ensure study integrity:

5. Main Results or Findings:

6. Most important clinical implications:

DESIGN AND METHODS PERSPECTIVE

1. Which research design did this study use?

   ☐ Experimental ☐ Case-Control ☐ Cross-sectional ☐ Randomized Controlled Clinical Trial
   ☐ Cohort ☐ Correlational ☐ Other (specify)

2. Why do you believe the authors selected that design?

3. What was the primary outcome of interest being measured?

4. How was the primary outcome defined and measured, using what type of variable?

5. List the statistical methods used in the order they were used in the study.

6. Why did the authors use that approach to analysis?

7. Was the primary hypothesis supported or rejected?

8. What secondary outcomes or measurements/variables were studied? List up to two.

9. Were the secondary outcomes of significance or importance to the findings?

10. What are two limitations and two strengths of the study discussed by the authors?

11. What is the main result or finding?

12. What is the value of this research to the practice of medicine?

dAC/JI 72009© UNTHSC (v. 8_3_09)

Appendix II 20-item test

1. Statistical significance means
   a. The probability that observed differences are due to chance alone
   b. The likelihood of a manuscript being accepted by a high impact factor journal
   c. The difference is large and important
   d. Rejecting alternative hypothesis at 0.05 level of significance

2. With 30 subjects I found a "significant" improvement from baseline to end-point in my treatment group but not in my control group (15 in each). But I found no "significant" difference between groups at end point. What would most effectively control for this problem?
   a. Use a test robust for small numbers
   b. Match the groups
   c. Control for extraneous variables
   d. Increase the number of subjects

3. The sensitivity of a test is
   a. The probability that the test will identify the condition of interest when it is actually present
   b. The humane approach of a questionnaire to the sensibilities of the research participant
   c. The ability of a test to accurately identify a condition of interest to be "not present”
   d. The probability of true negatives of all negative cases in the population

4. Clinical trials are usually conducted in a series of steps, called phases. Which phase clinical trial does not have therapeutic objectives, but only estimates the safety and effects of dosage/procedures?
   a. Phase I
   b. Phase II
   c. Phase III
   d. Phase IV

5. The purpose of linear regression is to
   a. Determine how much performance scores decrease over time
   b. Analyze nominal variables only
   c. Analyze ordinal variables only
   d. A method that examines the relation of a dependent variable to specified independent variables

6. There is a reason to question the validity of a research report if the author does not provide
   a. Parameters for the general population
   b. Adjustments for noted confounding variables
   c. Limitations
   d. Specifics of design and methods used

7. Which one of the following laws governs the protection of human subjects in biomedical research in the United States?
    a. PL 93-348, National Research Act
    b. FDA Regulations on Good Clinical Practices
    c. International Conference on Harmonization
    d. DHHS Code of Regulations

8. A study to determine if fasting is associated with dengue fever collected data from 40 infected patients matched on age, gender, and race to 40 without dengue fever. Hospital charts were reviewed to determine whether the subjects fasted prior to the illness onset. This type of study is:
    a. Cross-sectional study
    b. Case-control study
    c. Concurrent cohort study
    d. Retrospective cohort study

9. A placebo controlled trial of aspirin and dipyridamole to prevent arterial restenosis after coronary angioplasty, 38% of subjects receiving the true drug had restenosis, and 39% of patients on placebo had restenosis. The only information you have is that the P value is greater than 0.05.
    a. This is not enough information to determine drug efficacy.
    b. There could be a clinical trend toward efficacy
    c. There is a 95% chance that the effects are likely due to the drug
    d. I need to know if the data met the assumptions for the statistical test used

10. Systematic error in a study that results in a mistaken estimate of the effect of an exposure on the risk of a disease is called what?
    a. Confounding
    b. Bias
    c. Interaction
    d. Stratification

11. To study cardiovascular deaths comparing a new drug to a placebo, I need 200 subjects in each group to detect a 15% difference in cardiovascular end points given 90% power and a significance level of 0.01. Which of the following changes would cause me to have to increase my sample size?
    a. Aim to detect a 20% difference
    b. Specify a power of 80%
    c. Use a significance level of 0.05
    d. Aim to detect a difference of 10%

12. In a research study, the age of the subjects was 26 years ± 5 years (mean ± standard deviation). Which statement is most correct?
    a. It is 95% certain that the true mean age lies within the interval of 16-36 years
    b. Most of the patients were aged 26, and the rest were between 21 and 31 years
    c. Approximately 95% of the patients were between 16 and 36 years
    d. No patients were younger than age 16 or older than 36

13. The prevalence of hypertension by gender is presented in this table for a county in Texas:

	**Hypertension**	
**Gender**	Yes	No
Male	75	446
Female	125	570

Referring to the hypertension data presented above, what is the method to determine if men's hypertension proportion differs from women's?

a. McNemar Odds Ratio
b. Prevalence rate
c. Chi-square
d. Analysis of variance

14. In your clinic you give 150 patients a new pain management drug. All the patients had muscle pain. You contact 140 of them and they report less pain with the new drug. You decide

a. The conclusion may be invalid because there is no control group.
b. The conclusion may be applied to all your patients.
c. The conclusion may be invalid because we don't know what other medications they may have taken
d. The study should be published.

15. In your clinic you tested 500 patients ages 18 - 65 with a newly developed kit for urinary tract infection (UTI). The results are given above. What is the positive predictive value for the new test kit for this group?

	**True State of UTI**	
**Test Result**	Positive	Negative
Positive	309	49
Negative	16	126

a. 309/(309+49)
b. (309+49)/(309+49+16+926)
c. (309)/(309+16)
d. (309+16)/(309+49+16+926)

Please identify the following types of variables as

e. discrete b) continuous c) nominal or d) ordinal

16. _____ Birth weight in grams
17. _____ Birth weight classified as low, medium or high
18. _____ Type of delivery classified as cesarean, natural or induced

19. As you explain a one-year Phase II clinical trial of a new medication for depression, a prospective study participant says she is hoping to start a family in the next few months. One of the side effects may be a rare birth defect but the chance is 1 in 1,000. You
    a. Tell the person she cannot qualify for the study
    b. Secure her agreement to not become pregnant during the study
    c. Enroll the subject, hoping she is randomized to the placebo group
    d. Tell her the risks and let her decide if she wants to participate anyway
    e. Tell her the risks and let her decide if she wants to participate anyway

20. To determine a sample size for a study of how energy levels change over time in children with wheat allergies on a high protein diet compared to those on a non restricted diet, I need to have
    a. An estimate of the change I expect to see in my treatment group, the budget, the time schedule for the study, and the inclusion criteria
    b. The effect size from a similar or previous study, the power I want the study to have, the research design, and the confidence level I am willing to accept
    c. The details of the diet, the way I am measuring energy, the age group to be included in the study
    d. Some estimate of the amount of change I want to detect between groups, a definition of "energy level", and the ages of the subjects

21. Answers: 1. A, 2. D, 3. A, 4. A, 5. D, 6. D, 7. A, 8. B, 9. A, 10. A, 11. D, 12. A, 13. C, 14. A & C, 15. C, 16. B, 17. D, 18. C, 19. A, 20. B

NOTE: Each question also contained a "No Response" choice.
